# Association between coronary thrombus composition and microvascular obstruction following percutaneous coronary intervention in patients with acute myocardial infarction: A proof-of-concept study

**DOI:** 10.1097/MD.0000000000044639

**Published:** 2025-09-19

**Authors:** Mingliang Du, Hui Hui, Shize Sun, Lili Sun, Yunliang Sun, Junjun Zhao, Xiaoqun Zheng

**Affiliations:** a Department of Coronary Heart Disease, Central Hospital of Dalian University of Technology (Dalian Municipal Central Hospital), Dalian, Liaoning Province, China; b Department of Pathology, Central Hospital of Dalian University of Technology (Dalian Municipal Central Hospital), Dalian, Liaoning Province, China.

**Keywords:** acute myocardial infarction, coronary microvascular obstruction, coronary thrombus, immunohistochemical, percutaneous coronary intervention

## Abstract

Coronary microvascular obstruction (CMVO) significantly increases the incidence of major adverse cardiovascular events in patients with acute myocardial infarction (AMI). However, no studies to date have specifically examined the relationship between intracoronary thrombus composition and CMVO formation in AMI patients. This proof-of-concept study aims to investigate the association between coronary thrombus composition and CMVO during AMI. This study enrolled fifty-one patients diagnosed with acute ST-segment elevation myocardial infarction (STEMI) who underwent emergency percutaneous coronary intervention. Intracoronary thrombus specimens were systematically collected during the procedure. Based on established diagnostic criteria for CMVO, participants were stratified into 2 cohorts: the CMVO group exhibiting CMVO (n = 32) and the non-CMVO group without CMVO (n = 19). Histochemical profiling of coronary thrombi was performed to quantify inflammatory and vascular biomarkers including soluble CD40 ligand (sCD40-L), high-sensitivity C-reactive protein (hs-CRP), vascular cell adhesion molecule-1 (VCAM-1), endothelial cell-specific molecule-1 (Endocan), peptidoglycan recognition protein 1 (PGLYRP1), tumor necrosis factor receptor 1 (TNFR1), growth differentiation factor 15 (GDF15), TNF receptor superfamily member 10C (TNFRSF10C), and galectin-3.For quantitative assessment, 2 to 5 random microscopic fields were selected in both the peripheral and core regions of each thrombus specimen. The percentage of positively stained areas was calculated using digital image analysis software (ImageJ, NIH), with mean values derived from all examined fields for intergroup comparisons. Statistical analyses were conducted using SPSS 26.0 with significance threshold set at *P* < .05. Histochemical quantification showed differential expression of inflammatory mediators in percentage of positively stained areas: sCD40-L, hs-CRP, and VCAM-1 were markedly elevated in thrombi from CMVO patients. Multivariate logistic regression incorporating significant univariate predictors demonstrated, Biomarker analysis revealed significant positive correlations: sCD40-L: B = 0.540, *P* = .001; hs-CRP: B = 0.264, *P* = .007; VCAM-1: B = 0.281, *P* = .013. In addition,the CMVO group (CMVO-positive) demonstrated elevated mean levels of door-to-wire time, Low-Density Lipoprotein Cholesterol (LDL-C), body mass index, and serum interleukin-6 (IL-6) compared to controls. Quantitative analysis of coronary thrombus components revealed significant positive correlations between sCD40-L, hs-CRP, VCAM-1 levels and CMVO prevalence. Moreover, systemic metabolic disturbances - particularly type 2 diabetes mellitus, elevated serum LDL-C concentrations and heightened IL-6 levels were identified as independent predisposing factors for CMVO development.

## 1. Introduction

Acute myocardial infarction (AMI), characterized by the sudden and complete occlusion of the coronary arteries, leads to severe and persistent ischemia and necrosis of the myocardium. This condition is a critical manifestation within the spectrum of acute coronary syndromes, characterized by high rates of mortality, disability, and generally poor prognosis. Currently, the primary treatment for AMI is percutaneous coronary intervention (PCI), which aims to reperfuse the myocardium. However, the effectiveness of this intervention is notably compromised by inadequate microvascular perfusion following reperfusion of the epicardial large vessels. A phenomenon known as coronary microvascular obstruction (CMVO) occurs when there is reestablishment of flow in the epicardial coronary arteries but myocardial reperfusion remains impaired. Evidence indicates that after PCI, despite re-opening of the culprit vessel, over 50% of patients exhibit signs of microvascular damage on cardiac magnetic resonance imaging.^[1]^ In the absence of adequate reperfusion, the infarct core may evolve into irreversible myocardial hemorrhage, increasing the incidence of early complications of myocardial infarction, exacerbating ventricular remodeling, and notably elevating both hospitalization and mortality rates.^[2]^

Multiple mechanisms contribute to coronary microvascular obstruction, including ischemia-reperfusion injury, formation of microemboli, myocardial cell edema, inflammation, and cardiovascular risk factors. Among these, microemboli formed from thrombotic debris play a crucial role in the onset of CMVO. Selected biomarkers were chosen based on prior evidence linking them to platelet activation (sCD40-L), systemic inflammation (hs-CRP), and endothelial dysfunction (VCAM-1) – all of which are implicated in CMVO pathogenesis. The study focused on inflammatory and endothelial biomarkers within coronary thrombi, aiming to elucidate their potential roles in microvascular injury.

Despite the recognition of thrombus-derived microemboli as contributors to CMVO, the specific biological components of aspirated thrombi and their potential association with microvascular obstruction remain insufficiently characterized. By applying quantitative immunohistochemical analysis directly to aspirated thrombus specimens, this study overcomes the spatial and cellular resolution limitations associated with serum biomarker assays and noninvasive imaging modalities, enabling precise localization and quantification of these mediators at the thrombus–microvasculature interface and providing mechanistic insight into thrombus-driven microvascular obstruction. Therefore, the primary objective of this study was to compare the expression profiles of selected biomarkers in thrombi from AMI patients with and without CMVO, in order to explore thrombus-driven mechanisms of microvascular injury.

## 2. Materials and methods

### 2.1. Study population

This study consecutively enrolled 51 patients admitted for acute ST-segment elevation myocardial infarction (STEMI) who underwent emergency PCI from January 2023 to June 2024 in Central Hospital of Dalian University of Technology (Dalian Municipal Central Hospital). Inclusion criteria encompassed: participants were required to be aged 18 to 79 years; diagnosis of acute STEMI according to established clinical guidelines^[3]^; no history of myocardial infarction; emergency coronary angiography revealing acute single-vessel occlusion; thrombus aspiration performed during PCI with retention of thrombus specimens for analysis. Exclusion criteria were: patients who did not undergo emergency PCI; cardiac arrest, cardiogenic shock, hypersensitivity to contrast agents or allergic diathesis, or refusal to sign the informed consent; Advanced kidney dysfunction (creatinine clearance below 30 mL/min/m^2^ or receiving renal replacement therapy); prolonged treatment with immunosuppressive drugs or ongoing immune system suppression. Approval for the study came from the Medical Ethics Committee of Central Hospital of Dalian University of Technology (Dalian Municipal Central Hospital), under the reference number YN2022-039-35.

### 2.2. Clinical and laboratory data

The electronic medical record system was used to compile initial data on patients, covering age, sex, body mass index (BMI), personal history, comorbidities, and medical history (hypertension, diabetes), as well as door-to-balloon time (D to B time). Preoperative and postoperative clinical laboratory parameters were extracted, including high-sensitivity troponin (hs-TnT), hemoglobin, creatinine, N-terminal pro-Brain Natriuretic Peptide (NT-proBNP), low-density lipoprotein cholesterol (LDL-C), and IL-6. Data on preoperative dual antiplatelet therapy and postoperative echocardiographic assessment of left ventricular ejection fraction (LVEF) were also recorded.

### 2.3. Immunohistochemical procedures

**Figure 1.**
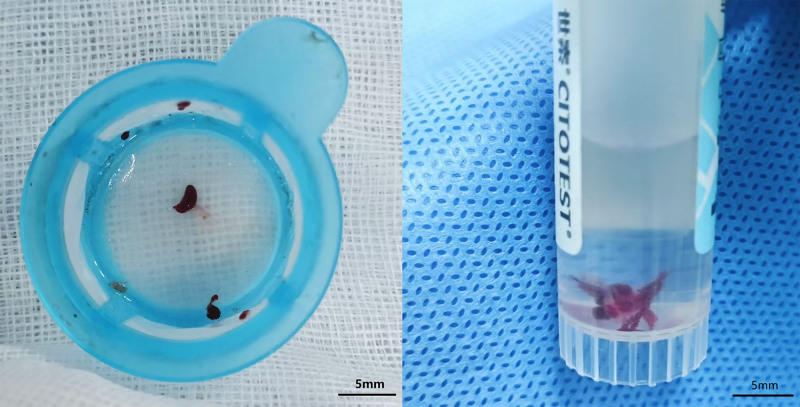
Coronary artery thrombus specimens were collected during the PCI. PCI = percutaneous coronary intervention

(1) In a surgical setting, thrombi were first preserved in 10% neutral buffered formalin for 48 hours. Post-preservation, thrombi were dehydrated through a graded ethanol series (70%, 80%, 90%, and 2 immersions in 100% ethanol; 1 minute each), then embedded in paraffin. From these paraffin blocks, sections of 3 µm in thickness were cut, refer to Figure 1.

(2) The sections were incubated at 60°C for 15 to 30 minutes, treated with xylene I and xylene II for 15 to 30 minutes each, followed by successive immersions in 100% alcohol I and alcohol II for 2 minutes each. This process was followed by sequential dehydration in 95%, 80%, and 70% alcohol for 1 minute each, rinsing with tap water, and washing with PBS. The samples were immersed in 0.01M citrate buffer at a pH of 6.0. They were heated to boiling in a microwave for 5 minutes before being allowed to cool to ambient temperature. The samples were then rinsed 3 times using PBS. To inhibit endogenous peroxidase, the samples were exposed to a 3% hydrogen peroxide solution for 5 minutes and subsequently rinsed with PBS again. For permeabilization, the samples underwent a 30-minute incubation in 1% Triton X-100, followed by a PBS wash and a 1-hour block in 5% BSA.

Primary antibody incubation was performed overnight at 4 °C using rabbit polyclonal antibodies (dilution 1:250) with the following specifications:

- CD40-L (Jiangsu Qingke Biological Research Center Co., Ltd.; DF2301)
- hs-CRP (Jiangsu Qingke; DF6027)
- Renalase (Jiangsu Qingke; DF6027)
- VCAM-1 (Jiangsu Qingke; DF6082)
- Endocan (Wuhan Yunkelong Technologies Co., Ltd.; UPAC463Hu01)
- PGLYRP1 (Wuhan Yunkelong; UPAD937Hu01)
- Tumor necrosis factor receptor 1 (Jiangsu Qingke; AF0282)
- GDF15 (Jiangsu Qingke; DF4096)
- TNFRSF10C (Jiangsu Qingke; DF6271)
- Galectin-3 (Jiangsu Qingke; AF0164)

Subsequently, the samples were rinsed with PBS the following day before a secondary antibody was applied and left to incubate at room temperature for an hour. After another PBS wash, the samples were processed with diaminobenzidine (DAB) solution to visualize the staining.

Negative controls were performed by omitting the primary antibody and using only the secondary detection reagent to confirm absence of nonspecific staining.

Counterstaining was performed with hematoxylin for 5 minutes, followed by a series of dehydration steps in 70%, 80%, 95%, and 100% alcohol for 1 minute each, and clearing in xylene I and xylene II for 5 minutes each. The slides were finally mounted with neutral gum.

Image acquisition was performed on a Nikon Ti-S microscope (MK2025073003SCI) at 200 × magnification.

(3) Microscopic examination revealed positive staining as brown-yellow cytoplasmic granules. For each sample, 2 to 5 random fields at both the periphery and core were scored. Two independent operators – blinded to group allocation – performed optical density measurements using ImageJ software, and the mean value was reported as the final result. Inter-rater consistency was assessed by calculating the intraclass correlation coefficient.

Renalase immunostaining followed the same protocol as other markers, with its inclusion justified by literature linking Renalase expression to CMVO pathogenesis.

### 2.4. Research methodology

According to the “Chinese Expert Consensus on the Diagnosis and Treatment of Coronary Microvascular Disease (2023 Edition),”^[4]^ the criteria include:

(1) Post-PCI myocardial infarction thrombolysis treatment (thrombolysis in myocardial infarction, TIMI) flow grading of 0 to 2.

(2) Post-PCI TIMI myocardial blush grading of 0 to 2.

(3) A decrease in ST-segment elevation amplitude of <50% 90 minutes post-surgery.

(4) Predischarge imaging findings indicating no myocardial perfusion in target areas using single-photon emission computed tomography, and first-pass perfusion defects or delayed gadolinium enhancement in cardiovascular magnetic resonance imaging (CMR).

Patients were classified into 2 groups: the CMVO group (CMVO group, n = 32) and the group with normal microcirculatory function (non-CMVO group, n = 19).

### 2.5. Statistical analysis

Data analysis was conducted using SPSS version 26.0. Normality was assessed using the Shapiro–Wilk test. Continuous variables that followed a normal distribution were expressed as mean ± standard deviation, and comparisons between groups were made using the t-test. For data not normally distributed, the median (P25, P75,where P25 and P75 represent the 25th and 75th percentiles, respectively) was used for representation, and the Mann–Whitney *U* test was employed for intergroup comparisons. Categorical data were expressed as counts (percentage), and the chi-square test was utilized for comparisons between groups. Multivariable logistic regression analysis was applied to assess the independent factors influencing CMVO. Variables with a *P* <.05 in univariable analysis were included in the multivariable model. No LSD–*t*-test was applied, since only 2‐group comparisons were performed. Statistical significance was defined with a *P*-value under .05. The Nagelkerke R² for the final logistic regression model was 0.536.

Quantitative analysis of immunohistochemical staining was performed using ImageJ software. Social history (smoking) and medical history (hypertension, diabetes) were analyzed as separate covariates. All quantitative data were presented as mean ± standard deviation (Mean ± SD), unless otherwise specified. GraphPad Prism version 9.0 was used for data visualization and supplementary statistical analysis.

## 3. Results

The primary objective of this study was to compare the expression profiles of selected biomarkers in thrombi from AMI patients with and without CMVO.

### 3.1. General baseline data

Compared with the non-CMVO group, the CMVO group differed significantly in 6 baseline variables: prevalent diabetes, door-to-wire (D to W), LDL-C, BMI, serum IL-6 levels, and mean LVEF (all *P* < .05). The proportion of individuals with diabetes was notably higher in the CMVO group compared to the non-CMVO group. Similarly, the CMVO group showed notably higher mean levels of D to W, LDL-C, BMI, and serum IL-6, while the mean LVEF percentage was notably lower. There were no significant differences in sex, preoperative antiplatelet therapy, culprit vessel, hypertension, smoking status, or age (*P* > .05), as shown in Table 1.

**Table 1:** Analysis of differences in general data between the 2 groups

	Parameters		Non-CMVO group(n = 19)	CMVO group (n = 32)	T/X^2^ Value	*P*-value
Demographics	Sex	Male	15 (78.95)	23 (71.88)	0.052	.820
		Female	4 (21.05)	9 (28.13)		
	Age (year-old)		55.37 ± 11.67	58.81 ± 13.18	-0.941	.352
	BMI (kg/m²)		25.1 ± 4.13	27.23 ± 2.99	-2.132	.038
Social history	Smoking	No	11 (57.89)	16 (50)	0.298	.585
		Yes	8 (42.11)	16 (50)		
Medical history	Hypertension	No	12 (63.16)	12 (37.5)	3.150	.076
		Yes	7 (36.84)	20 (62.5)		
	Diabetes	No	16 (84.21)	16 (50)	5.969	.015
		Yes	3 (15.79)	16 (50)		
Treatment	Preoperative antiplatelet therapy	Clopidogrel	7 (36.84)	11 (34.38)	0.032	.859
		Ticagrelor	12 (63.16)	21 (65.63)		
Angiographic Characteristics	Culprit vessel	LAD	7 (36.84)	16 (50)	1.605	.529
		LCX	2 (10.53)	5 (15.63)		
		RCA	10 (52.63)	11 (34.38)		
PCI-related	D to W (min)		64.63 ± 23.67	80.34 ± 23.28	-2.316	.025
Biomarkers	LDL-C (mmol/L)		2.88 ± 0.93	3.51 ± 0.75	-2.650	.011
	Serum IL-6 (mmol/L)		6.75 ± 4.05	25.23 ± 27.99	-3.672	.001
	LVEF %		53.21 ± 6.57	48.28 ± 7.54	2.363	.022

BMI = body mass index, CMVO = coronary microvascular obstruction, D to W = door-to-wire, IL-6 = interleukin-6, LAD = left anterior descending, LCX = left circumflex branch, LDL-C = low-density lipoprotein cholesterol, LVEF = left ventricular ejection fraction, PCI = percutaneous coronary intervention, RCA = right coronary artery, T/X² = t-test/Chi-square value.

### 3.2. Preoperative and postoperative comparative analysis between groups

Preoperatively, hemoglobin (HB), serum creatinine, NT-proBNP, and troponin T did not differ significantly between groups (*P* > .05). Postoperatively, the CMVO group had significantly lower HB than controls (132.96 ± 14.66 vs 145.88 ± 12.52 g/L, *P* = .033). There were no significant postoperative differences in creatinine, NT-proBNP, or troponin (*P* > .05), as indicated in Table 2.

**Table 2:** Intergroup analysis of various indicators

Time point	Parameters	Non-CMVO group (n = 19)	CMVO group (n = 32)	T Value	*P*-value
Preoperative	HB(g/L)	154.86 ± 17.32	147.24 ± 14.55	1.105	0.281
	Serum creatinine (mmol/L)	61.57 ± 10.63	76.86 ± 37.03	-1.062	0.300
	NT-proBNP(pg/mL)	225.43 ± 314.49	732.14 ± 1629.21	-0.806	0.429
	hs-TnT(ng/mL)	0.16 ± 0.19	1.7 ± 3.33	-1.900	0.075
Postoperative	HB(g/L)	145.88 ± 12.52	132.96 ± 14.66	2.230	0.033
	Serum creatinine (mmol/L)	72.08 ± 9.71	73.65 ± 30.62	-0.158	0.876
	NT-proBNP (pg/mL)	839.43 ± 596.25	1812.08 ± 1531.67	-1.628	0.114
	hs-TnT(ng/mL)	3.7 ± 3.61	6.29 ± 4.58	-1.538	0.133

CMVO = coronary microvascular obstruction, HB = hemoglobin, hs-TnT = high-sensitivity troponin, NT-proBNP = N-terminal pro-brain natriuretic peptide.

### 3.3. Intragroup comparison of preoperative and postoperative indicators

Within the non-CMVO group, postoperative HB decreased (145.33 ± 12.47 vs 160.00 ± 11.75 g/L, *P* = .004), and NT-proBNP (840.4 ± 559.9 vs 60.8 ± 31.28 pg/mL, *P* = .034) and troponin T (3.59 ± 3.09 vs 0.17 ± 0.21 ng/mL, *P* = .044) increased significantly; serum creatinine remained unchanged (*P* = .344). In the CMVO group, postoperative HB decreased (138.33 ± 12.67 vs 149.00 ± 14.01 g/L, *P* = .002) and troponin T increased (7.26 ± 4.85 vs 1.75 ± 3.43 ng/mL, *P* = .001); changes in creatinine and NT-proBNP were not significant (*P* < .05), as detailed in Table 3.

**Table 3:** Intragroup analysis of various indicators.

Groups	Parameters	Preoperative	Postoperative	T value	*P*-value
Non-CMVO group (n = 19)	HB(g/L)	160 ± 11.75	145.33 ± 12.47	5.108	.004
	Serum creatinine (mmol/L)	65.33 ± 4.08	70.5 ± 11.27	−1.045	.344
	NT-proBNP (pg/mL)	60.8 ± 31.28	840.4 ± 559.9	−3.177	.034
	hs-TnT(ng/mL)	0.17 ± 0.21	3.59 ± 3.09	−2.685	.044
CMVO group (n = 32)	HB (g/L)	149 ± 14.01	138.33 ± 12.67	3.786	.002
	Serum creatinine (mmol/L)	76.86 ± 37.03	79.02 ± 35.05	−0.428	.675
	NT-proBNP (pg/ml)	742.28 ± 1685.87	1683.13 ± 1755.83	−2.126	.052
	hs-TnT (ng/mL)	1.75 ± 3.43	7.26 ± 4.85	-4.008	.001

CMVO = coronary microvascular obstruction, HB = hemoglobin, hs-TnT = high-sensitivity troponin, NT-proBNP = N-terminal pro-brain natriuretic peptide.

Data are presented as Mean ± SD. Statistical comparisons were performed using paired t-tests.

### 3.4. Comparative analysis of immunohistochemical outcomes between two groups

**Figure 2.**
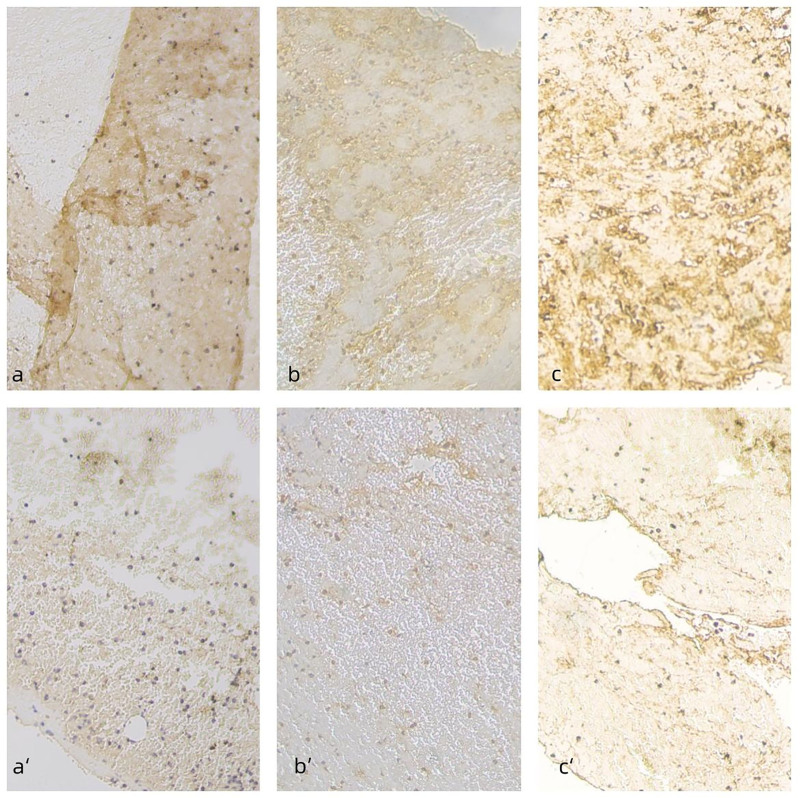
Immunohistochemical staining images of thrombi for sCD40-L, hs-CRP, VCAM-1 in sequential sections. Panels (A and B) and (C) represent immunostaining of sCD40-L, hs-CRP, VCAM-1 in the CMVO group, respectively, while panels a’, b’, and c’ correspond to the non-CMVO group. hs-CRP = high-sensitivity C-reactive protein, sCD40-L = soluble CD40 ligand, CMVO = coronary microvascular obstruction, VCAM-1 = vascular cell adhesion molecule-1

**Figure 3.**
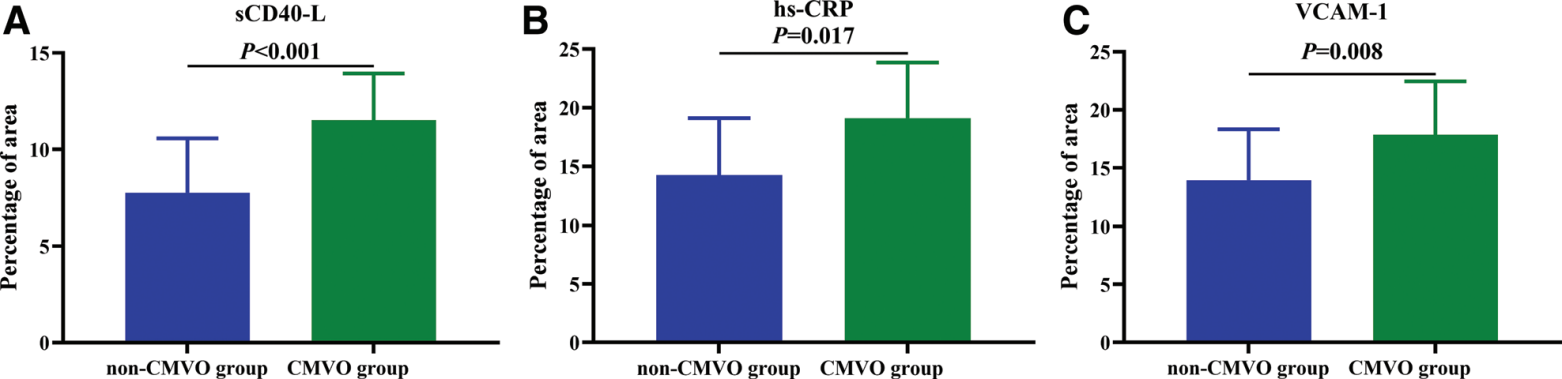
Bar graph illustrating the relationship between levels of sCD40-L, hs-CRP, VCAM-1 in coronary artery thrombi of the non-CMVO groups and CMVO groups. (The height of the column represents the mean and bar represents the standard deviation). hs-CRP = high-sensitivity C-reactive protein, sCD40-L = soluble CD40 ligand, CMVO = coronary microvascular obstruction, VCAM-1 = vascular cell adhesion molecule-1.

Significant between-group differences were observed for sCD40-L (11.51 ± 2.43 vs 7.75 ± 2.82, *P* < .001), hs-CRP (19.12 ± 4.74 vs 14.25 ± 4.87, *P* = .017), and VCAM-1 (17.85 ± 4.60 vs 13.93 ± 4.39, *P* = .008), while Endocan, PGLYRP1, tumor necrosis factor receptor 1, GDF15, TNFRSF10C, Galectin-3, and Renalase did not differ significantly (*P* > .05) (Table 4 and Figs. 2 and 3).

**Table 4:** Analysis of differences in immunohistochemical indicators between the 2 groups.

Parameters	Non-CMVO group (n = 19)	CMVO group (n = 32)	T Value	*P*-Value
Endocan	6.91 ± 3.52	8.25 ± 4.02	−1.204	.235
PGLYRP1	8.12 ± 3.88	10.01 ± 3.33	−1.842	.071
TNFR1	15.81 ± 5.19	13.27 ± 4.76	1.782	.081
sCD40-L	7.75 ± 2.82	11.51 ± 2.43	−5.032	.000
hs-CRP	14.25 ± 4.87	19.12 ± 4.74	−2.463	.017
GDF15	14.83 ± 5.82	14.21 ± 3.49	0.477	.636
TNFRSF10C	5.69 ± 3.53	7.68 ± 5.71	−1.369	.177
Galectin--3	13.23 ± 4.58	14.12 ± 3.73	−0.756	.453
Renalase	16.33 ± 4.49	16.03 ± 4.67	0.225	.823
VCAM-1	13.93 ± 4.39	17.85 ± 4.60	−2.786	.008

CMVO = coronary microvascular obstruction, GDF15 = growth differentiation factor 15, hs-CRP = high-sensitivity C-reactive protein, sCD40-L = soluble CD40 ligand, TNFR1 = tumor necrosis factor receptor 1, VCAM-1 = vascular cell adhesion molecule-1.

Data are presented as Mean ± SD. Statistical comparisons were performed using paired *t*-tests.

### 3.5. Logistic regression analysis using single significant indicators

A logistic regression model including variables with significant between-group differences identified diabetes (OR 2132.35, *P* = .037), LDL-C (OR 37.54, *P* = .039), and IL-6 (OR 1.83, *P* = .020) as independent predictors of CMVO (Table 5). Coefficients for sCD40-L, hs-CRP, and VCAM-1 were also positive and statistically significant (*P* < .05), indicating their association with increased CMVO risk (Table 5). The Nagelkerke R² for the logistic regression model was 0.536.

**Table 5:** Logistic regression analysis of factors influencing CMVO

Parameters	B	Standard deviation	Wald	*P*	Exp(B)	95% CI of Exp(B)
Diabetes	7.665	3.669	4.364	.037	2132.348	(2832706.449–2832706.449)
D to W	0.085	0.050	2.895	.089	1.089	(1.202–1.202)
LDL-C	3.625	1.758	4.253	.039	37.536	(1177.158–1177.158)
BMI	−0.009	0.212	0.002	.965	0.991	(1.501–1.501)
Serum IL-6	0.602	0.258	5.446	.020	1.825	(3.025–3.025)
LVEF %	−0.168	0.126	1.784	.182	0.845	(1.082–1.082)
sCD40-L	0.540	0.157	11.846	.001	1.717	(1.262–2.335)
hs-CRP	0.264	0.098	7.292	.007	1.302	(1.075–1.576)
VCAM-1	0.281	0.114	6.103	.013	1.324	(1.06–1.654)
Constant	−3.908	1.803	4.697	.030	0.020	–

95% CI = 95% confidence interval for exp(B), B = regression coefficient, BMI = body mass index, D to W = door-to-wire, CMVO = coronary microvascular obstruction, Exp(B) = exponentiated coefficient (odds ratio), hs-CRP = high-sensitivity C-reactive protein, LDL-C = low-density lipoprotein cholesterol, LVEF = left ventricular ejection fraction, sCD40-L = soluble CD40 ligand, VCAM-1 = vascular cell adhesion molecule-1, Wald = wald chi-square statistic.

## 4. Discussion

This study represents a novel approach by applying direct immunohistochemical quantification of thrombus components in AMI patients, allowing localized analysis of biomarker expression at the site of coronary occlusion – a major advancement over prior serum-based methods. To ensure robustness of the findings, intergroup variability was assessed using standard deviation and statistical comparisons, while intragroup variability was minimized through blinded dual-operator scoring and intraclass correlation coefficient validation. This study highlights the interplay between atherogenic status (e.g., elevated LDL-C), systemic inflammation (e.g., IL-6, hs-CRP), and coagulation-related endothelial dysfunction (e.g., sCD40-L, VCAM-1), which collectively contribute to CMVO pathogenesis.

The coronary arteries are categorized based on their diameter and function into 3 segments: epicardial large arteries with a diameter > 500µm, pre-small arteries with diameters between 100 and 500 µm, and small arteries with diameters < 100 µm. Collectively, the latter 2 are referred to as the coronary microcirculation, including microarteries, capillaries, and microveins. Under normal conditions, the resistance to coronary circulation from epicardial large arteries is typically <10%. Only when 70% of the luminal occlusion occurs does it notably affect coronary hemodynamics.^[5]^ It is established that 70% of the resistance to coronary circulation under physiological conditions is determined by the coronary microcirculation, which plays a crucial role in the physiological regulation of myocardial blood supply in response to changes in myocardial metabolic demand. In fact, at rest, the myocardial oxygen uptake is close to its maximum, and the delivery of oxygen to the myocardium almost entirely depends on the coronary blood flow reserve (CFR). In healthy individuals, any increase in myocardial oxygen demand can be met through coronary vasodilation and the adjustment of vascular resistance, allowing the coronary blood flow to increase up to 5 times the baseline level.^[6]^

This study confirms that diabetes, LDL-C, and serum IL-6 levels are influential factors in the occurrence of CMVO. Diabetes is a strong contributing factor to CMVO, but this relationship is bidirectional, as microvascular dysfunction in muscle and adipose tissue can also promote the onset of diabetes.^[7]^ Hypercholesterolemia notably reduces CFR, and its contribution to CMVO is associated with impaired endothelium-dependent vasodilation of small coronary arteries, possibly due to increased production of reactive oxygen species. Research has shown that in patients with CMVO following myocardial infarction, serum IL-6 levels are notably elevated, and early administration of tocilizumab (an IL-6 receptor antagonist) can reduce the area of myocardial infarction.^[8]^ This medication is well-tolerated with no major safety concerns, however, the clinical significance of this study remains uncertain, necessitating further large-scale research to explore the impact of tocilizumab on clinical endpoint events. Targeted therapy is still in the exploratory phase, and the safety and efficacy of treatments for CMVO require further clinical trial support.

Current research has established that the dislodgment of microemboli is a critical pathogenic mechanism leading to coronary microcirculatory obstruction during AMI. Microemboli are primarily composed of platelet aggregates, fibrin, amyloid, and atherosclerotic plaque material. In experimental models (pound dogs), when microemboli obstruct 50% of the coronary microvasculature, baseline perfusion levels of myocardial cells begin to decline.^[9]^ Microvascular embolism is typically accompanied by an inflammatory response, damaging neighboring viable myocardial cells through signaling pathways involving nitric oxide, tumor necrosis factor (TNF), sphingosine, reactive oxygen species, and the myofibrillar oxidative signaling system.^[10]^ This finding is crucial because even a minor formation of microemboli during emergency PCI in AMI patients, which does not affect myocardial perfusion levels, can activate signaling pathways leading to the release of pro-platelet aggregation factors (such as serotonin, thromboxane A2, and tissue factor), vasoconstrictors (such as endothelin-1, thromboxane A2, and tissue factor), and substances related to endothelial dysfunction (such as TNF), all of which can intensify the degree of coronary microcirculatory obstruction.

In the findings of this study, mean levels of sCD40-L, hs-CRP, and VCAM-1 were notably higher in thrombi from patients with CMVO compared to those without CMVO. Soluble CD40 ligand, a member of the TNF superfamily, is involved in inflammatory responses, platelet activation, and endothelial damage, and is associated with the instability of atherosclerotic plaques and thrombogenesis. By activating platelets and endothelial cells, sCD40-L promotes the release of pro-inflammatory cytokines (such as IL-6 and TNF-α), leading to microvascular spasms, thrombosis, and disruption of the endothelial barrier. Levels of sCD40-L can predict the occurrence of microvascular obstruction (MVO) and are positively correlated with the area of microcirculatory injury as indicated by CMR.^[11]^ Given that over 95% of sCD40L originates from platelets, it has been assumed that the level of sCD40L reflects platelet activation. Consequently, it is important to consider that the predictive ability of sCD40L in STEMI may be diminished by antiplatelet medications, as these drugs inhibit platelet aggregation and the release of sCD40L.^[12]^ hs-CRP exacerbates endothelial dysfunction and microvascular inflammation by activating the complement system, promoting monocyte chemotaxis, and enhancing oxidative stress. High mean levels of hs-CRP are notably associated with intramyocardial hemorrhage and microvascular obstruction detected by CMR, underscoring the central role of inflammation in microcirculatory injury.^[13]^ In the setting of AMI, inflammation and thrombosis seem to be closely related. Actually, hs-CRP may play a role as a local pro-inflammatory mediator during the acute phase of myocardial infarction by activating complement which can directly stimulate the coagulation cascade. In addition, hs-CRP alters the fibrinolytic balance of endothelial cells and therefore promotes intravascular fibrin formation.^[14]^ These complex and only partially understood interactions with platelets, the blood coagulation and the fibrinolytic system may further explain the association of hs-CRP and CMVO after STEMI. VCAM-1, a marker of endothelial cell activation, mediates leukocyte adhesion and migration, thus promoting vascular inflammation and microcirculatory disorders. In patients with STEMI, serum levels of VCAM-1 are positively correlated with the index of microcirculatory resistance. Elevated VCAM-1 can predict no-reflow phenomena post-reperfusion treatment, suggesting that endothelial activation is a critical factor in microcirculatory obstruction.^[15]^ Although systemic markers such as serum IL-6 were elevated in CMVO patients, and hs-CRP thrombus-level expression via IHC may reflect localized inflammatory activity at the site of vascular injury, suggesting a partial overlap but not complete concordance between systemic and local inflammation. While serum levels of VCAM-1 were not measured in this study, future research could explore correlations between circulating and thrombus-level expression.

Several limitations of our study need to be acknowledged. First, this is a single-center, proof-of-concept investigation with a modest sample size, which may limit generalizability. Second, the cross-sectional design precludes causal inference between thrombus composition and CMVO. Third, selection bias may have arisen from including only patients undergoing successful thrombus aspiration, potentially excluding those with inaccessible or minimal thrombus burden. Finally, we lacked longitudinal follow-up for major adverse cardiac events (MACE), preventing evaluation of long-term prognostic impact. Future multicenter, prospective studies with larger cohorts and standardized follow-up are needed to validate and extend these findings.

## 5. Conclusion

CMVO is a significant determinant of adverse outcomes in patients post-PCI for AMI. In this study, we demonstrated that diabetes, LDL-C, and elevated serum IL-6 levels serve as independent predictors of CMVO, and that thrombus expression of sCD40-L, hs-CRP, and VCAM-1 is closely associated with microvascular obstruction. Although numerous therapeutic strategies exist, most lack robust validation from large-scale randomized controlled trials, and their clinical utility remains to be established. Future research could further elucidate the direct roles of sCD40-L and VCAM-1 in microvascular obstruction through animal models, and investigate whether targeting inflammatory or endothelial pathways with drugs such as IL-1β inhibitors, anti-CD40-L antibodies, or IL-6 receptor inhibitors could improve outcomes in CMVO. Additionally, proactive management of related risk factors, such as type 2 diabetes and hypercholesterolemia, may further reduce the prevalence of CMVO. Importantly, future studies should be multicenter, randomized trials with long-term follow-up to assess MACE, and large prospective cohorts should evaluate whether intensive management of diabetes and hypercholesterolemia reduces CMVO prevalence and improves patient prognosis.

## Author contributions

**Conceptualization:** Mingliang Du, Hui Hui, Shize Sun, Lili Sun, Junjun Zhao, Xiaoqun Zheng.

**Data curation:** Mingliang Du, Hui Hui, Yunliang Sun, Junjun Zhao, Xiaoqun Zheng.

**Validation:** Mingliang Du.

**Visualization:** Mingliang Du.

**Writing** – **original draft:** Mingliang Du, Hui Hui, Xiaoqun Zheng.

**Writing** – **review & editing:** Mingliang Du.
